# Pharmacokinetic, biologic and epidemiologic differences in MPA- and NET-based progestin-only injectable contraceptives relative to the potential impact on HIV acquisition in women^☆^^☆☆^

**DOI:** 10.1016/j.contraception.2018.12.001

**Published:** 2019-04

**Authors:** Renee Heffron, Sharon L. Achilles, Laneta J. Dorflinger, Janet P. Hapgood, James Kiarie, Chelsea B. Polis, Petrus S. Steyn

**Affiliations:** a Department of Global Health, University of Washington, 325 Ninth Avenue Box 359927, Seattle, WA, USA; b Department of Epidemiology, University of Washington, 325 Ninth Avenue Box 359927, Seattle, WA, USA; c Department of Obstetrics, Gynecology, and Reproductive Sciences and Center for Family Planning Research, University of Pittsburgh School of Medicine, Pittsburgh, PA, USA; d Magee-Womens Research Institute, Pittsburgh, PA, USA; e FHI 360, 359 Blackwell Street, Suite 200, Durham, NC, USA 27701; f Department of Molecular and Cell Biology, Faculty of Science, University of Cape Town, Private Bag X3, Rondebosch, 7701, Cape Town, South Africa; g Institute of Infectious Disease and Molecular Medicine, Faculty of Health Sciences, University of Cape Town, Private Bag X3, Rondebosch, 7701, Cape Town, South Africa; h Department of Reproductive Health and Research, World Health Organization (WHO), Geneva, Switzerland; i Guttmacher Institute, 125 Maiden Lane, 7th Floor, Manhattan, New York, 10038, USA; j Department of Epidemiology, Johns Hopkins Bloomberg School of Public Health, 615 North Wolfe St., Baltimore, MD 21205, USA

**Keywords:** Contraception, DMPA, NET-EN, Progestin, HIV

## Abstract

Access to safe and effective contraceptive choices is a reproductive right and contributes tremendously to improvements in maternal and child health. Progestin-only injectables, particularly intramuscularly injected depot medroxyprogesterone acetate (DMPA-IM), have received increased attention given findings suggesting a potential association with increased HIV risk. For women at high risk of HIV, the World Health Organization's *Medical eligibility criteria for contraceptive use* currently aggregate recommendations for all progestin-only injectables, including DMPA-IM, subcutaneously injected DMPA (DMPA-SC) and intramuscularly injected norethindrone/ norethisterone enanthate (NET-EN), except in the case of some drug interactions. We considered whether published data indicate differences or similarities between these injectables relevant to risk of acquiring HIV. *In vitro* data confirm different biological activities of these distinct progestins, including that MPA, and not NET, binds and activates the glucocorticoid receptor resulting in different biological effects relevant to immune function. Limited clinical data suggest changes in immunologic activity following DMPA-IM and NET-EN initiation, but interstudy variation and study design differences diminish ability to determine clinical relevance and the degree to which DMPA-IM and NET-EN could act differentially. The highest-quality epidemiologic studies suggest a potential 40% increase in HIV incidence in users of DMPA-IM relative to women not using hormonal contraception but no significant increase in risk in users of NET-EN. In our opinion, most of the available biologic activity and epidemiologic data indicate that DMPA and NET-EN are likely to act differently, and data remain too limited to evaluate differences between DMPA-IM and DMPA-SC.

## 1 Introduction

Access to sexual and reproductive health services, including contraceptive choice, is fundamental to the rights and well-being of women and girls. Hormonal contraceptives including progestin-only injectables (intramuscular [IM] and subcutaneous [SC] depot medroxyprogesterone acetate [DMPA, henceforth DMPA-IM and DMPA-SC] and norethindrone/norethisterone enanthate [NET-EN]) are highly effective methods of pregnancy prevention [1], [2], [3]. The availability and effective use of these contraceptive methods decrease the occurrence of unintended pregnancies, pregnancy-related mortality and morbidity, and mother-to-child transmission of HIV, and also improves infant and child health [4], [5], [6].

The World Health Organization (WHO) has developed the *Medical eligibility criteria for contraceptive use* (MEC) to provide guidance to national family planning programs around the provision and use of contraceptive methods. The MEC provide a comprehensive set of recommendations on the safety of contraceptives with respect to specific medical conditions, characteristics and concomitant medication use by individual users [7]. WHO continuously monitors the literature for new evidence on contraceptive methods and regularly convenes experts to consider new data and update the MEC, as appropriate [7], [8], [9], [10], [11]. One important characteristic delineated within the MEC guidance is the use of contraceptives by women at high risk for sexually transmitted infections, including HIV. For almost 3 decades, research related to use of hormonal contraceptives and HIV acquisition risk in women has been published [12], [13], [14]. Progestin-only injectables, particularly DMPA-IM, have received the most attention due to a number of studies finding a potential increased HIV acquisition risk among users. Many of these studies have methodological limitations that complicate data interpretation, and thus, this area of work continues to be the subject of periodic review with expert consultation to aid in interpretation of the overall body of literature [10]. In 2016, the MEC category for progestin-only injectables for women at high risk of HIV was revised from a “1*” (indicating no restriction on use of the method but including an important clarification^1^) to a “2” (indicating that the advantages generally outweigh the theoretical or proven risks) and highlighted the need for more robust data [9].

DMPA-IM is one of three globally available progestin-only injectables, all of which prevent ovulation and thicken cervical mucus as a means to prevent pregnancy. DMPA-IM is a three-monthly intramuscular injection of 150 mg of MPA [2]. It is the most commonly used hormonal contraceptive in many sub-Saharan African countries [15]. DMPA-SC is three-monthly subcutaneous injection of 104 mg of MPA and has similar contraceptive efficacy to DMPA-IM [1]. DMPA-SC is available in a single-use autodisable device and can be either self-injected or delivered by a community health worker, where policy allows, as well as by clinician. As the newest injectable contraceptive (first approval was in 2004 [16]), DMPA-SC has regulatory approval in >40 countries and is available in 15 countries currently [17], [18]. NET-EN is a three-monthly intramuscular injection of 200 mg of NET that has similar contraceptive efficacy to DMPA-IM when delivered according to the recommended two-monthly schedule [3].

The current WHO MEC have the following categories for contraceptives: (1) combined (i.e., estrogen plus progestin) hormonal contraceptives (which aggregate pill, patch, ring and combined injectables), (2) progestogen-only pills, (3) progestogen-only injectables (aggregating DMPA-IM, DMPA-SC and NET-EN), (4) implants, (5) levonorgestrel intrauterine device (IUD) and (6) copper IUD [7]. The MEC have disaggregated guidance for methods within a category when data to support refinements are available. For example, recommendations for progestin-only injectables (i.e., DMPA and NET-EN) are currently disaggregated specifically for women using certain anticonvulsants, rifampicin/rifabutin and certain antiretroviral therapies [7]. Participants at a WHO-convened meeting in August 2017 reviewed and discussed the current available evidence, including pharmacokinetic, biologic and epidemiologic data, related to DMPA-IM, DMPA-SC and NET-EN to consider whether recommendations for progestin-only injectables should be similarly disaggregated for women at high risk of HIV within the MEC. From that meeting, the authors of this manuscript were asked to review data on differences between DMPA-IM, DMPA-SC and NET-EN. The objective of this commentary is to present the synthesis of these data and to identify important research gaps.

## 2 Pharmacologic, biologic and epidemiologic studies of DMPA and NET-EN and HIV risk

We read studies across the fields of pharmacology, *in vitro* and clinical studies with biologic outcomes, and longitudinal epidemiologic studies with HIV outcomes. For each area, we discussed findings and limitations from key studies that shaped our interpretation of the data. Each of the subsequent sections presents our collective opinion on the status of the field, including key insights and gaps, and we highlight key studies that influenced our opinion.

### 2.1 Pharmacologic profiles

The pharmacokinetic data from studies of DMPA and NET-EN show large interindividual and interstudy variability. Differences across studies may be impacted by differences in study design, including numbers of women investigated, women's demographic characteristics (ethnicity, race, body mass index, weight and metabolism), number of injections, injection sites, and time and frequency of measurements [14], [19]. Assay methodologies to measure progestins have evolved over time to be more sensitive and specific; however, this can further complicate interpretation when comparing data from newer and older studies [20], [21], [22]. Because of the pharmacokinetics of the formulations, serum concentrations of MPA and NET in the first weeks following injection of DMPA-IM and NET-EN, respectively, exceed contraceptive efficacy but are necessary to ensure maintenance of contraceptive efficacy throughout the dosing duration. For DMPA-IM, contraceptive efficacy has been shown to be the same with 100 mg and 150 mg of DMPA-IM [1], [2], [3], [23]. At the prescribed doses, DMPA has a longer duration of action than NET-EN. NET and MPA bind differentially to serum proteins, resulting in different bioavailability of free active drug [24].

No pharmacokinetic studies have directly compared DMPA-IM and DMPA-SC. However, data from non-head-to-head studies suggest that serum MPA concentrations may be greater during the first 30 days after injection of DMPA-IM relative to DMPA-SC [19], [25], [26], [27]. More than 30 days following injection, serum MPA concentrations appear similar in women receiving DMPA-IM and DMPA-SC injections, although comparisons are complicated by lack of a head-to-head study [19], [25], [26], [28]. A postmarketing study designed to compare efficacy, safety and acceptability of DMPA-SC with DMPA-IM suggested no difference in minimum concentration (*C*_min_) at 90 days following injection, even subsequent to multiple injections, but did not evaluate *C*_max_ [29].

With regard to pharmacodynamic activity, DMPA-IM, DMPA-SC and NET-EN all suppress ovulation for the prescribed duration. DMPA-IM and NET-EN appear to have similar effects on weight gain [30] and duration of bleeding and spotting, although DMPA-IM users are more likely to develop amenorrhea compared to women using NET-EN [31], [32]. Return to ovulation may be somewhat more rapid with NET-EN compared to DMPA-IM [33], yet the mean time to fertility is likely similar postinjection in users of NET-EN compared to DMPA-IM users [34], [35]. In two randomized clinical trials, side effects of DMPA-IM and DMPA-SC were similar with regard to weight gain and frequency of amenorrhea, while skin irritation and injection site adverse events were slightly more common among DMPA-SC users [26], [29], [36]. DMPA-IM and DMPA-SC are known to reduce bone mineral density, and NET-EN may slow the growth of bone mineral density in young women still attaining peak bone mass [29], [37], [38].

### 2.2 Data on biologic mechanisms potentially relevant for HIV risk

Numerous studies have investigated several potential mechanisms by which DMPA-IM and/or NET-EN could alter HIV acquisition risk, including steroid receptor activity and the relationship of steroid properties to adaptive and innate immunity, HIV cellular targets, microbiota, genital tract barrier function and tissue architecture. To date, multiple clinical studies of biological mechanisms have evaluated DMPA-IM, few studies have similarly investigated NET-EN, and no studies have evaluated DMPA-SC. Results from these clinical studies are mixed, and their interpretation is challenging due to uncertain contraceptive exposures (self-reported contraceptive use and method switching) and to aggregation of “injectable” without distinguishing progestin type [39], [40], [41], [42], [43], [44], [45], [46], [47], [48], [49], [50], [51]. Multiple in vitro studies have been conducted using the specific progestins MPA and NET, including various concentrations, and although these studies show differences in cellular responses, these differences may or may not translate to the organismal level. Thus, numerous questions remain unanswered.

#### 2.2.1 Steroid receptor binding and activity

Multiple *in vitro* investigations have evaluated the steroid receptor binding activity of MPA and NET. MPA binds to and activates progesterone, glucocorticoid and androgen receptors, whereas NET binds to and activates progesterone and androgen receptors [52], [53], [54], [55]. MPA appears to have a higher binding affinity for and activity via the progesterone receptor compared to NET [54]. MPA and NET have similar affinities for and activities via the androgen receptor [56]. MPA binds and activates the glucocorticoid receptor with high affinity, while NET binds with such low affinity that it is likely not clinically relevant [14], [52], [53], [54], [55]. The affinity of MPA for the glucocorticoid receptor is reportedly greater than that of cortisol, an immune-modulating hormone and the natural ligand for the glucocorticoid receptor [57]. MPA-stimulated glucocorticoid activity exhibits dose–response characteristics *in vitro* [24], [58]. Furthermore, metabolites of both of these drugs could modulate *in vivo* activity [55], [59], [60]. For example, in women, NET has been shown to be converted to ethinyl estradiol, a potent estrogen, which binds to and activates the estrogen receptor [59]. For DMPA, further clinical studies are needed to determine whether different routes of administration (e.g., IM versus SC) or sites of subcutaneous injection (e.g., abdomen, thigh, arm) result in different receptor-mediated responses [19], [25], [26], [28], [54], [55], [61]. The differential binding affinity of MPA and NET for the glucocorticoid receptor may underlie key differences in biological impacts [52], [54], [55] including potential differences in HIV susceptibility [14], [24].

#### 2.2.2 Steroid properties related to adaptive and innate immunity

Glucocorticoid binding to its ubiquitous receptor can stimulate or inhibit transcription of multiple target genes, which may subsequently elicit changes in several physiologic responses, some of which have been linked to HIV susceptibility, including changes in cytokine levels and immune cell activity [52], [53], [54], [55]. *In vitro*, physiologically relevant concentrations of MPA alter expression levels of several cytokine and chemokine genes [14], [24], [53], [62], [63], [64], [65] and transcription via promoter elements [58]. In *in vitro* studies of peripheral blood mononuclear cells, exposure to MPA decreases cytokine production [24], [44], [65], [66], including interferon-α and tumor necrosis factor-α, and impaired function of dendritic cells [40], [65]. *In vitro*, MPA also increases T-cell apoptosis at relevant contraceptive concentrations [67], increases activation of immune cells and increases expression of the CCR5 HIV coreceptor [68] and HIV infection [64], [68], [69]. The glucocorticoid receptor has been shown to be involved in several of these *in vitro* MPA effects [24], [53], [62], [63], [67], [68]. Parallel studies show that NET has no effect on these functions compared to MPA *in vitro* [24], [53], [58], [63], [65], [67], [68].

In clinical studies with varying designs and robustness, DMPA-IM users exhibited increased proinflammatory cytokines and immune activation measured in genital samples [39], [46], [47], [50], [70], while NET-EN users exhibited similar but less proinflammatory effects compared to DMPA-IM users [46]. Other studies have shown decreased proinflammatory cytokines and chemokines in women using various injectable progestins compared to women not using hormonal contraception [44], [48], and one study has shown no change in proinflammatory cytokines in women using DMPA-IM compared to women not using hormonal contraception [49]. Thus, results from the body of clinical literature assessing the effects of DMPA-IM on immune function are inconsistent, and specific data for NET-EN are limited. Given the data demonstrating in vitro effects of MPA, but not NET, on glucocorticoid activity and immune function, these studies predict that if NET affects immune function in women, this is likely mediated via mechanisms other than those involving the glucocorticoid receptor. Definitive conclusions about the potential for different effects of DMPA and NET-EN on immune function in vivo cannot be made at this time. Given the complexity of the innate and adaptive human immune system, extrapolation of data from in vitro to in vivo studies is challenging. While many of the effects of MPA observed in vitro have also been demonstrated in women on DMPA-IM [14], [42], [44], [48], [71], other clinical studies have had conflicting results [14], [49]. Future clinical studies to address the effects of contraceptives on specific biologic mechanisms need to be designed robustly to test the impact of specific markers while minimizing the influence of all other external factors.

#### 2.2.3 Cellular targets for HIV transmission

Results from clinical studies of DMPA-IM and NET-EN examining their impact on HIV target cells are inconsistent, and study designs vary greatly [39], [41], [71], [72]. No clinical data on cellular targets are currently published for NET-EN (except in aggregate with DMPA-IM) or DMPA-SC users. There may be differences between DMPA-IM and NET-EN with respect to their impact on the activity, frequency and function of systemic and genital tract HIV target cells *in vivo*. Some studies of tissue samples from women have found cervical HIV target cells to be more prevalent in the presence of higher systemic progestogen (including luteal phase progesterone or injectables), while other clinical studies have reported no differences in target cell quantities among DMPA-IM users relative to women not using contraception [42], [43], [44], [45]. One study of South African women using injectable progestins (76% using DMPA and 24% using NET-EN but analyzed as a single group) compared to naturally cycling women using no long-term contraceptive reported increased frequency of HIV target cells (3.92 times higher, p=.02) in injectable users compared with naturally cycling women [41].

#### 2.2.4 Microbiota

Vaginal dysbiosis, which may be associated with genital inflammation, has been implicated in a possible mechanistic pathway increasing HIV susceptibility [73], [74]. One study showed decreased quantities of H_2_O_2_-producing *Lactobacillus* species in DMPA-IM users relative to women not using contraception, which could result in increased propensity for bacterial vaginosis (BV) [70]. In contrast, one longitudinal study reported no decrease in BV-associated bacteria with DMPA-IM use relative to condom use [75], and a second study showed that women initiating DMPA-IM or NET-EN experienced no changes in beneficial *Lactobacillus* species, BV or quantities of key BV-associated organisms (*Gardnerella vaginalis, Atopobium vaginae* and *Megasphera)* [51]. A systematic review concluded that hormonal contraceptives (including progestin-only contraceptives) do not increase the risk of incident BV [76], which is consistent with a meta-analysis concluding that DMPA-IM has no detrimental effect on and possibly decreases the incidence of BV by 18%–30% [77]. Based on the strength of evidence, neither DMPA-IM nor NET-EN appears to shift microbiota towards clinically meaningful vaginal dysbiosis. There are no data examining vaginal microbiota changes with DMPA-SC use.

#### 2.2.5 Tissue architecture and genital tract permeability

Recent studies indicate that the epithelial layer may be altered after DMPA-IM initiation based on changes seen in *ex vivo* evaluation of epithelial permeability as well as observed changes in tight junction markers and epithelial repair proteins in clinical studies [39], [70], [72]. This is consistent with studies showing that physiologically relevant concentrations of MPA decrease barrier function *in vitro* [78]. Hormonal contraceptive use (specifically, DMPA-IM and combined oral contraceptives) in the presence of vaginal infections may result in a weakened mucosal barrier [49]. No studies to date have looked at NET-EN or DMPA-SC and architectural changes.

### 2.3 Epidemiologic data

Recent systematic reviews [12], [79], [80] have considered all available longitudinal studies estimating associations between use of specific methods of hormonal contraception and risk of HIV acquisition and have identified the subset of studies considered the most methodologically sound (although all currently available data are observational and thus have inherent limitations). A meta-analysis of these higher-quality studies which specifically examined the risk of HIV acquisition with use of DMPA-IM in comparison to nonuse of hormonal contraception (i.e., use of nonhormonal methods or nonuse of contraception) generated a hazard ratio of 1.40, which was statistically significant [95% confidence interval (CI): 1.23–1.59] [12]. A meta-analysis of these higher-quality studies assessing NET-EN use in comparison to nonuse of hormonal contraception generated a hazard ratio of 1.15 (95% CI 0.93–1.42), which was not statistically significant [12]. No studies to date have calculated HIV risk estimates for women using DMPA-SC relative to women using nonhormonal contraception or no method. Other meta-analyses have found similar results [81], [82], [83], although the design and quality of these meta-analyses varied, and all meta-analyses of observational data are vulnerable to the potential for spurious precision [84].

Two head-to-head comparisons of HIV risk among women using DMPA-IM versus NET-EN found statistically significant hazard ratios of 1.3–1.4, indicating a potential 30%–40% increase in HIV risk for DMPA-IM users versus NET-EN users [81], [85]. However, it is important to acknowledge that women choosing DMPA-IM and those choosing NET-EN may also have important sociodemographic differences (age, marital status, education, sexual behaviors, condom use, etc.) which could also differentially impact HIV risk and cannot be completely rectified by analytic techniques, including those used in these studies [85], [86]. Given that observational data have inherent limitations, the current WHO guidance states that uncertainty remains about whether any hormonal contraceptive method actually causes increased HIV risk and calls for higher-quality studies, including randomized clinical trials [7].

## 3 Discussion

Our review has identified important differences between MPA and NET with respect to sex steroid receptor binding affinities and their exhibition of different biological effects *in vitro* that may plausibly result in differential impact on HIV susceptibility in women. Data from epidemiologic studies are also suggestive of a difference between DMPA-IM and NET-EN with potentially increased HIV risk for DMPA-IM users (relative to women using no contraception or nonhormonal contraception) — but not for NET-EN users — and show significantly higher HIV risk for DMPA-IM users relative to NET-EN users in head-to-head comparisons which attempted to control for sociodemographic differences between DMPA-IM and NET-EN users. Results from clinical studies remain inconsistent without a clear overall conclusion to questions of how DMPA-IM and NET-EN could differentially alter pathways relative to HIV susceptibility, and this body of evidence is currently limited by suboptimal methodology and inconsistency across study designs. There are no data available on DMPA-SC relevant to HIV risk in women or mediating biological pathways, representing a large gap in the body of evidence.

Based on our overall review of these data, we find considerable data that could inform and support disaggregation of DMPA and NET-EN in contraceptive guidance for women at high risk of HIV. The available data suggest that these two biochemically different progestins have different biological effects, and epidemiologic data suggest different associations with HIV risk. Additional clinical data from high-quality studies are needed to definitively assess biological impacts that may alter HIV risk elicited by initiation and use of DMPA-IM, DMPA-SC and NET-EN as well as subsequent clinical outcomes, including HIV acquisition. There are no comparative data on DMPA-IM and DMPA-SC to support disaggregation of guidance for DMPA by route of delivery (IM versus SC) at this time. Further research should explore potential differences in these two routes of administration, and future clinical studies on injectable contraceptives should record specific progestin dose and content, formulation, route of administration and resulting progestin serum concentration. Further research should also consider a systems biology approach to explore the impact of mechanistic hypotheses on multiple physiologic processes, including function of the immune system.

In alignment with WHO recommendations to improve the quality of evidence examining associations of hormonal contraception and HIV risk, a large randomized trial of three highly effective contraceptives [DMPA-IM, levonorgestrel (LNG) implant and copper IUD] is ongoing with incident HIV as the primary outcome (clinicaltrials.gov #NCT02550067). Results may offer insight into HIV risk with DMPA-IM use relative to use of LNG implant or copper IUD. This trial will not inform HIV acquisition risk for women using any of the trial contraceptives relative to women using no contraception, pregnant women or women using NET-EN, DMPA-SC or other contraceptive methods.

Multiple research gaps in this area remain (Table 1). Additional data will contribute to more complete recommendations on each contraceptive method with regard to HIV risk. When designing *in vitro*, clinical and epidemiologic studies to address these specific gaps, it is critical to develop study designs and methods that are sufficiently rigorous to improve upon existing evidence. More recent clinical studies implemented objective categorization of specific progestin exposure, an improvement upon the plethora of studies that have relied on self-reported use of contraceptives [20], [22], [87]. Higher-quality studies would have longitudinal study designs, be powered to answer the primary study question, incorporate careful assessment of hormonal milieu and take place in geographic locations of differing HIV burden. These elements of higher quality are also needed for studies of other novel contraceptive technologies, although here we have limited our discussion to progestin-only injectables.

**Table 1:** Research gaps in understanding effects of DMPA-IM, DMPA-SC and NET-EN on HIV risk

• Determine the relative HIV incidence among women using DMPA-IM, DMPA-SC and NET-EN
• Determine the biological impacts of contraceptives relative to drug pharmacology — systemically and locally — with respect to potential mediators of HIV susceptibility, including:
○ Modulation of the microbiome
○ Modulation of HIV target cell recruitment, immune markers including cytokine and chemokine release, and impacts on adaptive and innate immune function
○ Alterations in tissue permeability and the relative impact on target cell recruitment and/or HIV penetration
○ Dose responses and differences in cellular mechanisms of action of MPA and NET
• Determine pharmacokinetic differences between DMPA-SC and DMPA-IM and evaluate biologic mechanisms to address whether DMPA-SC may differ from DMPA-IM with respect to HIV risk, especially in the first 30 days after injection and with repeated dosing and drug accumulation.

## 4 Conclusions

In our opinion, most of the available biologic activity and epidemiologic data indicate that DMPA and NET-EN are likely to act differently, and data remain too limited to evaluate differences between DMPA-IM and DMPA-SC.

## Footnotes

1 “Some studies suggest that women using progestogen-only injectable contraception may be at increased risk of HIV acquisition, other studies do not show this association. A WHO expert group reviewed all the available evidence and agreed that the data were not sufficiently conclusive to change current guidance. However, because of the inconclusive nature of the body of evidence on possible increased risk of HIV acquisition, women using progestogen-only injectable contraception should be strongly advised to also always use condoms, male or female, and other HIV preventive measures. Expansion of contraceptive method mix and further research on the relationship between hormonal contraception and HIV infection is essential. These recommendations will be continually reviewed in light of new evidence.”
